# Physiopathology of intratendinous calcific deposition

**DOI:** 10.1186/1741-7015-10-95

**Published:** 2012-08-23

**Authors:** Francesco Oliva, Alessio Giai Via, Nicola Maffulli

**Affiliations:** 1 Department of Orthopaedics and Traumatology, University of Rome 'Tor Vergata' School of Medicine, Viale Oxford 81, Rome, Italy; 2 Centre for Sports and Exercise Medicine Queen Mary University of London, Barts and The London School of Medicine and Dentistry, Mile End Hospital, 275 Bancroft Road, London, E1 4DG, UK

**Keywords:** Calcific Tendinopathy, Calcific Deposits, Tendons, Review

## Abstract

In calcific tendinopathy (CT), calcium deposits in the substance of the tendon, with chronic activity-related pain, tenderness, localized edema and various degrees of decreased range of motion. CT is particularly common in the rotator cuff, and supraspinatus, Achilles and patellar tendons. The presence of calcific deposits may worsen the clinical manifestations of tendinopathy with an increase in rupture rate, slower recovery times and a higher frequency of post-operative complications. The aetiopathogenesis of CT is still controversial, but seems to be the result of an active cell-mediated process and a localized attempt of the tendon to compensate the original decreased stiffness. Tendon healing includes many sequential processes, and disturbances at different stages of healing may lead to different combinations of histopathological changes, diverting the normal healing processes to an abnormal pathway. In this review, we discuss the theories of pathogenesis behind CT. Better understanding of the pathogenesis is essential for development of effective treatment modalities and for improvement of clinical outcomes.

## Introduction

**Figure 1:**
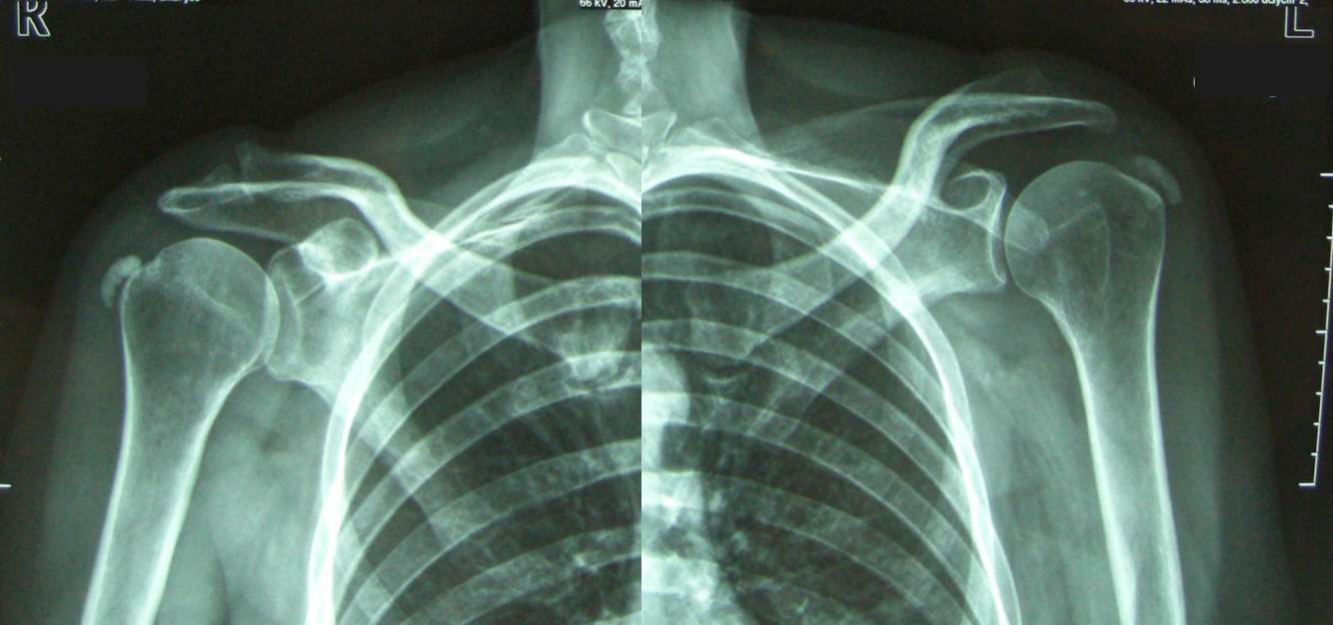
**Bilateral calcific tendinopathy (CT) of the shoulder**.

In calcific tendinopathy (CT) calcium deposits in the substance of the tendon. CT is particularly common in the rotator cuff tendons (RCTs) and supraspinatus tendon, and Achilles tendon and patellar tendon. CT of the rotator cuff is common in Caucasian populations, with a reported prevalence of 2.7% to 22%, mostly affecting women between 30 and 50 years. The most frequently involved tendon is the supraspinatus tendon, and in 10% of patients the condition is bilateral (Figure 1) [1]. The nomenclature of this condition is confusing, and, for example, in the shoulder terms such as calcific periarthritis, periarticular apatite deposition, and calcifying tendinitis have been used [2]. We suggest to use the terms 'calcific tendinopathy', as it underlines the lack of a clear pathogenesis when the process is located in the body of tendon, and 'insertional calcific tendinopathy', if the calcium deposit is located at the bone-tendon junction.

**Figure 2:**
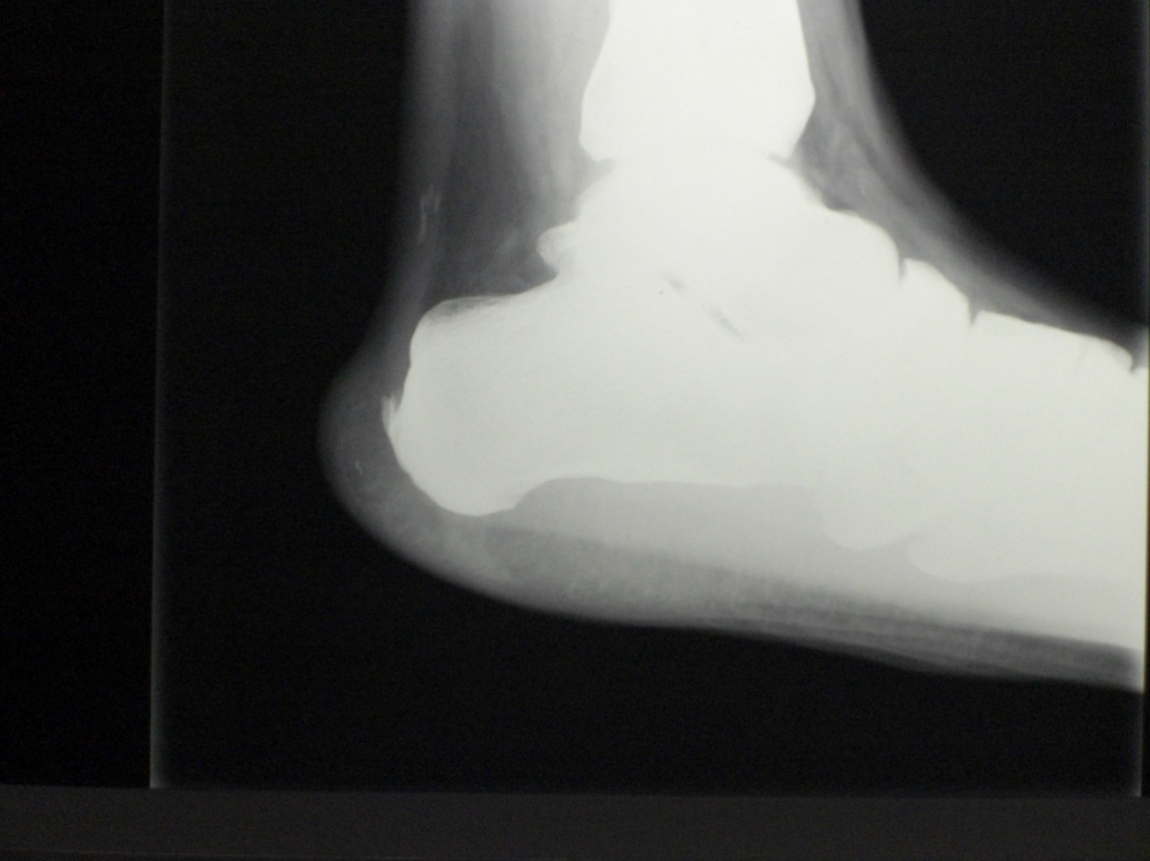
**Insertional calcific tendinopathy (CT) of the Achilles tendon associated with CT of the main body of the tendon**.

Calcific insertional tendinopathy of the Achilles tendon manifests in different patients populations, including young athletes and older, sedentary and overweight individuals [3]. Usually, radiographs evidence ossification at the insertion of the Achilles tendon or a spur (fish-hook osteophyte) on the superior portion of the calcaneus. CT in this location is often associated with retrocalcaneal bursitis or Haglund's deformity. The incidence of insertional tendinopathy of the Achilles tendon is not clear. The incidence varies from 5% to the most common presentation in athletes [3]; calcifications of the main body of the tendon are at best uncommon (Figure 2). CT of the patellar tendon is rare, and most patients with patellar tendinopathy show no evidence of ossification [4].

Most of the current treatment modalities are neither effective nor evidence-based because of our poor understanding on the underlying pathogenesis of CT. We review the present knowledge on this topic to stimulate further research.

### **Clinical manifestation**

Clinical manifestations of the calcific process within the tendons include chronic activity-related pain, tenderness, localized edema and various degrees of decreased range of motion (ROM). CT of the rotator cuff shows a tendency toward spontaneous resorption of the deposits and symptoms often resolve spontaneously, although some authors described persistent pain at long time follow-up and persistent reduction of ROM [5,6]. Osteolysis of the greater tuberosity is an uncommon and distinctive form of CT of the shoulder, and is associated with significantly poorer clinical and functional outcome both before and after surgical treatment [7]. Microscopic calcifications which are not detectable at plain radiography can also occur in chronic tendinopathy. A histological study showed high incidence of small calcium deposits in tendinopathic supraspinatus tendons [8]. Microscopic calcium deposits are frequent also in diabetic patients [9].

The clinical course of insertional CT has been poorly investigated, although experience suggests that pain seems to improve in older patients even though the insertional calcification persists. Generally, the presence of calcific deposits worsens the clinical manifestations of tendinopathy with an increase in rupture rate, slower recovery times and a higher frequency of post-operative complications [10].

### Historical review

As previously discussed, the nomenclature of CT is confusing, perhaps in part because of the many terms classically used to describe localized deposits of calcium in tendons [11], including calcifying tendinitis, calcific tendinitis, calcified tendinitis, calcareous tendinitis, tendinosis calcarea, calcific periarthritis, periarticular apatite deposit [10]. Some of them emphasise the extra-articular location of the deposit, others mention the nature of the compound found in the calcification or the process that might explain its deposition.

At the beginning of the 1950s, it was clear that local degeneration of the involved tendon precedes the deposition of calcium salts [11], and that there may be a constitutional predisposition.

Sandstrom, in 1938, speculated that necrosis of the tendon secondary to local ischemia and vascular changes was the first step to promote deposition of calcified material [12]. Bishop believed that repetitive minor trauma could induce rupture of the fibers of the supraspinatus tendon, hyaline degeneration and the deposition of calcium in the injured tendon [13]. The hypothesis was later supported by Bosworth and colleagues [14]. Urist *et al*. and Uhthoff *et al*. suggested the occurrence of an initial cartilage metaplasia of the tendon, followed by an active multifocal and cell-mediated calcifying process [15,16]. Recently, Mohr and Bilger described the process as beginning with the necrosis of tenocytes with concomitant intracellular accumulation of calcium, often in the form of microspheroliths or psammomas [17].

### Mineral components

Specimens of RCTs obtained during surgery consist of a gritty mass of sandy material or a toothpaste-like fluid, and the deposits were described as a white amorphous mass composed of many small round or ovoid bodies. Later, X-ray diffraction and infrared spectrometry and other techniques identified the material of calcific deposits as calcium carbonate apatite [18-20]. Computed tomography studies of patellar tendon revealed the three-dimensional structure of calcific deposits, which have a porous structure throughout the tendon [18]. Few investigations have been performed on the role of the types of carbonated apatite, although they have been reported to be a single component in the calcific deposits [21]. Two different types of carbonate apatite compose the calcific deposits, according to the position which carbonate ions (CO_3_^2-^) occupy in the hydroxyapatite (HAP). They are defined as A-type carbonate apatite and B-type carbonate apatite [22]. Gartner et al. [23] observed that the macroscopic differences of calcific deposits were not reflected in the mineralogical structure, and neither chemical compositional change nor a change in the crystal lattice was observed. They stated that no chemical dissolution process of the inorganic material was responsible for the resorption activity in the acute phase. More recently Chiou et al. studied the chemical components in CT of the RCTs, and they observed that different quantities of A- and B-type carbonated apatite changed in the formative, resting and resorption phases [24]. They noted reduced amounts A-type carbonate apatite and increased amounts located in the B positions during the process of progressive calcification. The same Authors classified calcific depositions into four shapes according to ultrasonographic findings: an arc shape, a fragmented or punctuated shape, a nodular shape, and a cystic shape. They also found a statistically significant association between the morphology of the calcified deposits and the clinical symptoms of the affected shoulder (Table 1).

**Table 1:** Correlation between morphology, progressive calcification process and symptoms of the mineral components of calcific tendinopathy (CT) of the rotator cuff, proposed by Chiou [24].

Calcific stage	Phase	Morphologic shape of calcific deposition	Clinical symptoms
Pre-calcific stage	Correlation not evaluated		
Calcific stage	Formative phase	Arc or fragmented/punctuated	Mild pain
	Resting phase	Nodular	Moderate to severe pain
	Resorptive phase	Cystic	Severe pain
Post-calcific stage	Correlation not evaluated		

Recently, matrix vesicles have been isolated in mature porcine patellar tendons [25]. Matrix vesicles are small extracellular organelles which are involved in mineralization of the extracellular matrix in many tissues, including bone and cartilage [26]. Previous studies demonstrate the presence of matrix vesicles near calcific deposition of the RCTs [16,27], and recently they have been isolated also in the extracellular matrix of normal patellar tendons [25]. The authors pointed out the importance of extracellular matrix vesicles in pathogenetic mechanism of CT. In the normal matrix, the vesicles are inhibited from mineralizing, but, in pathological conditions, such as injuries or matrix degeneration secondary to age or diabetes, they may be permitted to mineralize.

### Theories of pathogenesis

The aetiopathogenesis of CT is largely unknown, especially because it remains difficult to clarify the steps which induce crystal deposition into the tendon. Furthermore, biopsies of the pathologic RCTs are obtained only towards the end of the natural history of the condition, when patients are symptomatic [16]. Many different theories have been developed (see also Table 2).

**Table 2:** Different theories about etiopathogenesis of calcific tendinopathy (CT).

Type	Cause	Reference(s) by first author	Year(s)
Degenerative calcification	Vascular ischemia	Sandstrom [12]	1938
	Repetitive trauma	Bishop [13] and Bosworth [14]	1939, 1941
	Necrosis of tenocytes and intracellular calcium accumulation	Mohr [17]	1990
Reactive calcification	Active cell mediated process	Uhthoff [27]	1997
Endochondral ossification	Endochondral ossification of fibrocartilage at the enthesis of the tendon	Benjamin [34]	2000
Chondral metaplasia	Erroneous differentiation of tendon-derived stem cells (TDSCs)	Rui [46]	2011

### Reactive Calcification

Uhthoff and coworkers hypothesized that a favorable environment permits an active process of cell-mediated calcification, usually followed by spontaneous phagocytic resorption [28]. They describe four stages in the calcifying process of the rotator cuff: precalcific phase, calcific phase, resorptive phase, and repair phase. All phases may occur concomitantly in the same tendon. The precalcific stage involves fibrocartilaginous metaplasia within the tendon. In the second stage, the formative phase, calcific deposits are formed. This stage is subdivided into three phases: formative, resting, and resorptive. Calcium crystals are deposited primarily in matrix vesicles that coalesce to form large foci of calcification separated by chondrocytes and fibrocartilaginous tissue septae. The resting phase occurs when fibrocollagenous tissue borders the foci of calcification, indicating termination of deposition. The resorptive phase is marked by the appearance of thin-walled vascular channels at the periphery of the deposit. Macrophages and multinucleated giant cells then surround the deposit and phagocytose debris with calcium removal. In this phase, the deposit exhibits a thick, creamy, or toothpaste-like material that is often under pressure. The final stage involves an attempt by tendon to heal. Nakase et al. [29] clarified the nature of the multinucleated cells located near the calcium deposits. These were positive for cathepsin K [29], showing a typical osteoclast phenotype. Cathepsin K is a protease, it is a member of the peptidase C1 protein family, is predominantly expressed in osteoclasts, and it is involved in bone remodeling and resorption [30]. Some years after these proposals were made, osteopontin was observed in cells surrounding tendon calcifications [31,32]. Osteopontin is a member of the small integrin-binding ligand N-linked glycoprotein glycoprotein (SIBLING) family first identified in 1986 in osteoblasts. It plays important roles in many physiological and pathological processes, including wound healing and bone remodeling [33], but its role in CT has not been clarified [32].

### Endochondral Ossification

Achilles and patellar tendon calcifications are formed by a process resembling endochondral ossification, with bone formation and remodeling mediated by population of osteoblasts and osteoclasts.

Benjamin and coworkers proposed a model of insertional Achilles CT based on a rat study [34]. They suggested that insertional CT could develop by endochondral ossification of fibrocartilage at the enthesis of the Achilles tendon. The calcific process begins in the enthesis and grows into the tendon. Fibrocartilage cells appear by metaplasia from tenocytes. The fibrocartilage is then eroded, and blood vessels invade the rows of fibrocartilage cells from the underlying bone marrow. Finally, bone is deposited and the spur is formed. No inflammatory cells or microtears were identified. The authors believe that the increased surface at the tendon-bone junction may represent an adaptive mechanism to increased mechanical loads.

Lui et al. studied the histological features of collagenase-induced patellar tendon ossification in a rat model [35]. Many chondrocyte-like cells and the absence of infiltration of inflammatory cells were observed around the calcific deposits. They found a marked loss of collagen type I and an increase of collagen type II and type X, which occurred mainly at the chondrocyte-like cells and their surrounding matrix in the calcific deposits. Collagen type II is typical of cartilage and fibrocartilage, and it is resistant to compressive stresses. Type × collagen is a short chain collagen which has been associated with calcific cartilage and/or the expression of the hypertrophic chondrocyte phenotype. It is a marker of endochondral ossification. The same authors subsequently described an increased expression of collagen type III and a high collagen type III/collagen type I ratio [36]. The increase of collagen type III coincided with thinner, less organized and weaker tendon. Histological specimens of insertional calcific tendinopathy of Achilles tendons showed a greater intensity of staining for collagen type III than normal tendons [37], and higher than normal expression of collagen types III mRNA was detected high in human Achilles tendinopathy [38]. Chondrocyte markers were also evidenced in the clinical samples of calcific insertional Achilles tendinopathy and in rotator cuff tendinopathy [37,39].

### Chondral Metaplasia

Other authors thought that ectopic bone derives from metaplasia of tendon cells into osteogenic cells. Injection of recombinant human bone morphogenetic protein-2 (rhBMP-2) [40] into the tendon increased ectopic bone formation, indicating that the tendon consisted of cells that were responsive to BMP [41] and were capable of differentiating along the chondro-osseous pathway.

Mesenchymal stem cells are present in tendon tissues [42]. Human and mouse tendons hold cells with universal stem cell characteristics which could differentiate into chondrocytes and osteoblasts [43]. Rui et al. isolated Tendon-Derived Stem Cells (TDSCs) from the flexor tendon and patellar tendon of rats [44,45]. They proposed that chondral metaplasia and ectopic ossification may be caused by erroneous differentiation of tendon cells [46].

Which condition or stimulus is able to cause this erroneous differentiation of TDSCs? Many proteins could be involved in tendon degeneration, calcification and rearrangement processes, playing different roles in the various phases of calcification and resorption. Among the possible candidates are bone morphogenetic proteins (BMPs) and transglutaminases (TGs). Recently, Zang and Wang suggested that BMP-2-mediated effects on human TDSCs may contribute to the formation of calcific deposits in CT [47]. We observed an increased expression of osteopontin, cathepsin K and TG2 mRNA in the calcific areas of the supraspinatus tendon as compared to what observed in the normal tissue [48]. TG2 is ubiquitously expressed, and plays a role in a variety of cellular processes, including the crosstalk between macrophages and apoptotic cells, glucose tolerance and other processes. It is also important in maintaining the structural integrity of tendons and it could be involved in tendon repair [49]. The increased expression of osteopontin and TG2 could thus be compatible with their increased production in the calcific area, probably by osteoclast-like cells involved in the resorptive phase [29].

The mRNA and protein expression of major proteoglycans of extracellular matrix, including decorin, aggrecan, biglycan and fibromodulin and their relationship with ectopic chondrogenesis, ossification and loss of matrix organization, has been observed in a calcific tendinopathy model [36]. Decorin, aggrecan, biglycan and fibromodulin are small leucine rich repeated proteoglycans (SLRPs), which participate in collagen-fibril formation, and their expression patterns are altered in chronic tendinopathy [50]. Decorin is a component of connective tissue, binds to type I collagen fibrils, and plays a role in matrix assembly [51]. It is the most abundant SLRP found in tendon mid-substance. Aggrecan and biglycan are common in the fibrocartilaginous regions of the enthesis. Aggrecan plays an important role in the adaptation to compressive loads. Fibromodulin participates in the assembly of the extracellular matrix, and it interacts with type I and type II collagen fibrils [52]. A sustained or increased expression of decorin, aggrecan, biglycan and fibromodulin was found in this calcified tendinopathy model [36]. However, the presence of ectopic calcification in Achilles, patellar and quadriceps tendons was also reported in biglycan and fibromodulin knockout mice [53]. Another important feature of SLRPs is their ability to modulate the activity of the resident cell population by binding and sequestering growth factors [54,55]. The differentiation of tendon progenitor cells into chondrocytes and bone cells was modulated by the expression of biglycan and fibromodulin [56]. In normal tendon healing, TDSCs would proliferate and differentiate into tenocytes, but in particular conditions, they can differentiate into chondrocytes or osteoblasts, causing the deposition of the 'wrong' extracellular matrix and calcific deposits, resulting in failed healing and pain. The mechanism leading to the erroneous differentiation of TDSCs is not clear. It could be modulated by the expression of biglycan and fibromodulin, and by the expression of chondro-ostogenetic BMPs, such as BMP-2, BMP-4 and BMP-7, which were over-expressed in CT models [57]. Rui et al. therefore hypothesized that the erroneous differentiation of TDSCs into chondrocytes or osteoblasts instead of tenocytes could be the pathogenetic mechanism of calcifying tendinopathy [46]. Aberrant differentiation of stem cells has been postulated to be the cause of other disorders, including vascular calcification [58], skin calcification [59] and skeletal calcification [60].

### Predisposing Factors

An association between CT and diabetes and thyroid disorders has been shown, but the precise mechanism is still unknown [1]. Patients with associated endocrine disorders present earlier onset of symptoms, longer natural history, and they undergo surgery more frequently compared to a control population [61,62]. More than 30% of patients with insulin-dependent diabetes have tendon calcification [63]. The exposure of proteins to high levels of sugar moieties cause the glycosylation of several extra-cellular matrix proteins, which can modify the extracellular matrix by cross-linking proteins. In an animal study, tenocytes obtained from porcine patellar tendon have been incubated with glycated type I collagen, which increased Tg activity. This may represent an additional pathway mediating pathologic changes and could contribute to CT in diabetes [62].

**Figure 3:**
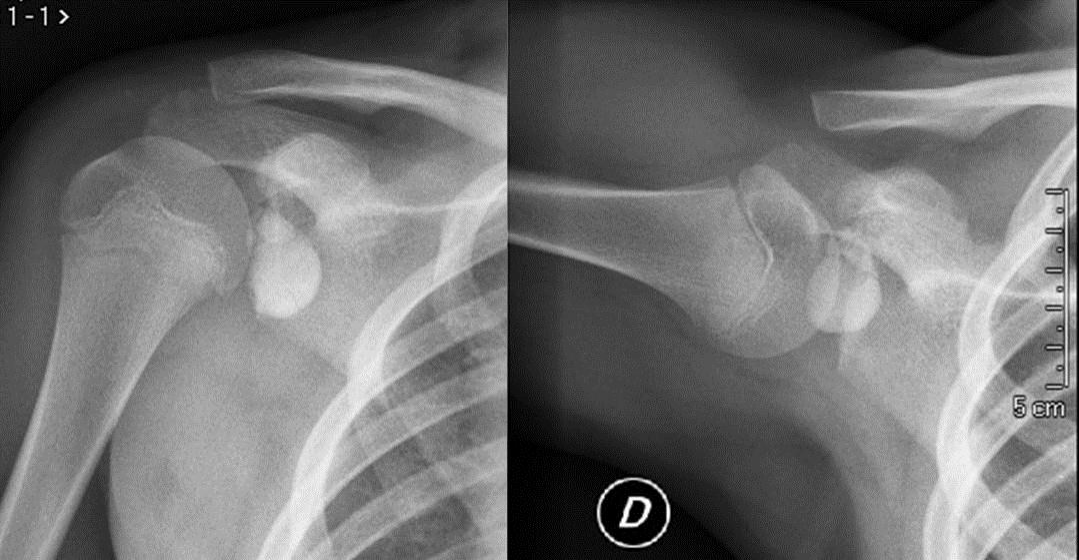
**Calcific tendinopathy (CT) of the subscapularis tendon in a 13-years-old boy**.

A familial predisposition and inherited genetic components have also been postulated as a cause of CT in some circumstances [64-67]. Variants within the COL5A1 [68], tenascin C [69] and matrix metalloproteinase 3 (MMP3) genes [70] are associated with increased risk of Achilles tendon injuries. CT of the RCTs have been observed in children, and hence it cannot be related to degenerative changes [71] (Figure 3). Therefore, some genetic variants could modify the susceptibility of tendons to the matrix abnormalities observed in tendinopathy [72].

## Discussion and Future perspectives

The aetiopathogenesis of CT is still controversial, especially because it remains difficult to clarify the first steps causing this condition and those involved in the development of calcifications.

Rather than being formed by precipitation of inorganic ions, CT results from an active cell-mediated process in which resident progenitor cells with multidifferentiation potential may play a determinant role [73].

**Figure 4:**
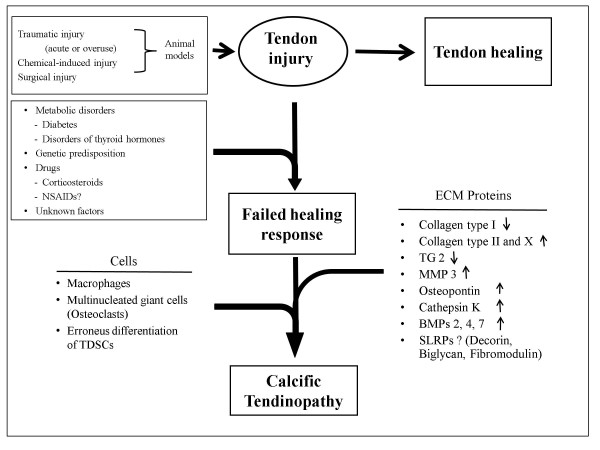
**The abnormal pathway that may lead to calcific tendinopathy (CT)**.

Many different factors such as acute injury, repetitive microtrauma, and chemical-induced injury may cause damage to the tendon and start the natural healing process. Tendon healing includes many sequential processes such as matrix synthesis and remodeling, synthesis of pro-inflammatory cytokines, neovascularization, neural modulations, recruitment of healing cells, multipotent cells, TDSCs, proliferation, apoptosis [74]. Disturbances at different stages of healing may lead to different combinations of histopathological changes. The normal healing processes are then diverted onto an abnormal pathway (Figure 4). Clinical features such as chronic pain, swelling, functional limitations and tendon ruptures are the consequences.

Since ossified tendons will have increased stiffness, ossification can be seen as a localized attempt to compensate for the original decreased stiffness of the weak tendon. It is possible that the erroneous differentiation of tendon progenitor cells into chondrocytes or osteoblasts instead of tenocytes may contribute to the pathogenesis of CT. The mechanism leading to the erroneous differentiation of TDSCs is not completely understood. Probably, the expression of BMPs, biglycan, fibromodulin and an unfavorable micro-environment induced by overuse modify the natural healing process of the tendon. Conservative management modalities such as non-steroidal anti-inflammatory drugs (NSAIDs) or corticosteroids are often prescribed, and may further influence the pathways of the failed healing [75]. NSAIDs could modulate tendon cell proliferation [76,77], the expression of extracellular matrix components [78] and degradative enzymes in cells culture studies [46]. Corticosteroids can induce a fibrocartilaginous phenotype in tendon cells [78], and induce osteogenic differentiation in human spinal ligament derived cells [79].

Many questions remain unanswered about the pathogenesis of CT. Calcium carbonate apatite appears the only component of calcific deposits, but inorganic component of Achilles and patellar CT has been less investigated than CT of the RCTs. Histological and imaging studies show that the three-dimensional structure of calcific deposits is quite different. Calcific depositions in the RCTs appear as a toothpaste-like fluid, while calcific deposits in the Achilles and patellar tendons have a porous structure [35] and a vascular core [34]. Therefore, we can speculate that their mineralogical structure could also be different.

'Calcific tendinopathy' and 'insertional calcific tendinopathy' are caused by two distinct pathogenetic mechanisms. In RCTs, degenerative changes in the extracellular matrix seem to play an important role for the formation of calcific deposits. The pathogenesis of CT involves matrix vesicles, macrophages and multinucleated giant cells with a typical osteoclast phenotype, producing a toothpaste-like material [25,29]. No vascular invasion has been documented. This process has not been observed in other tendons. Recently, Gohr et al. elucidated the role of matrix vesicles also in the patellar tendon [25], but, as the enthesis was removed, this model is more similar to a CT of the main body of the tendon than to an insertional CT. We also think that degenerative changes cannot be solely responsible, because we are not able to explain the deposition of calcium salt in twin brothers and children only with a reactive degenerative theory [65,66,71].

The mechanism of insertional CT has been clarified by Benjamin and coworkers, and essentially accepted worldwide [34] (Table 3). Increased vascularity in insertional Achilles tendinopathy was observed also by other authors [37].

**Table 3:** Pathogenetic models proposed for calcific tendinopathy (CT) of rotator cuff tendons (RCTs) and insertional CT of the Achilles tendon.

Stage	Cells	Extracellular matrix	Blood vessels	Inflammatory cells
CT model proposed by Uhthoff [28] for the rotator cuff:				
Pre-calcific stage	Chondrocyte-like cells	Fibrocartilaginous metaplasia, presence of calcium crystals collected into matrix vesicles	None	None
Calcific stage:				
Formative phase	Chondrocyte-like cells	Multifocal calcific deposits separated by fibrocollagenous tissue or fibrocartilage	None	None
Resting phase	Chondrocyte-like cells	Fibrocollagenous tissue borders the foci of calcification	None	None
Resorptive phase	Chondrocyte-like cells	Deposits of a thick, creamy, toothpaste-like material under pressure appear	Vascular channels near the calcific deposits	Macrophages and multinucleated giant cells cathepsin K-positive [22]
Post-calcific stage	Fibroblast	Granulation tissue, deposition of type III collagen	Capillaries surround the calcific deposits	Multinucleated giant cells [22]
Model proposed by Benjamin [34] for insertional CT of Achilles tendon:				
Early stage	Chondrocytes and fibrocartilage cells	Fibrocartilage metaplasia	None	None
Second stage	Few chondrocytes	Erosion of calcaneal cartilage. Fibrocartilage metaplasia of tendon matrix.	Blood vessels invade the calcaneal fibrocartilage	None
Bony spur formation	Few fibrocartilage cells	The original cartilage has disappeared	New bone is deposited around the blood vessels	None

CT of the rotator cuff has been investigated with histological studies of specimens obtained from human biopsies, while the study of CT of Achilles and patellar tendons is based on animal models of collagenase-induced tendinopathy. Therefore, we do not know whether the pathogenesis of CT of the rotator cuff can be compared to CT of the Achilles and patellar tendons. Moreover, no pathogenetic studies on the rotator cuff have been published since the late 1970s, and no animal CT studies are present in literature [80]. We do not know why the calcific deposits of the rotator cuff involve the main body of the tendon, while the most common presentation of CT in the Achilles tendon is insertional. Animal models of CT of RC seem necessary to understand its pathogenesis. No histological and epidemiological studies on CT of the main body of Achilles tendon are published.

Furthermore, no clinical or imaging classification has been published in the literature, except for the CT of RCTs [1]. Also, it is not clear whether the gross morphological anatomy of tendons (for example, the RC is a flat tendon, while the Achilles tendon is cylindrical) plays a role.

The involvement of mesenchymal stem cells (MSCs) in the pathogenesis of the CT process [46] and the role of autologous growth factors have been postulated, but not clarified [54,55].

While emerging data seem to indicate an association between tendinopathies and endocrine disorders such as diabetes, hypercholesterolemia, hypertriglyceridemia, thyroid disorders, and estrogen levels alterations [81,82], the association with CT is unclear, and no physiopathological investigations have been performed.

## Conclusions

The aetiopathogenesis of CT is still controversial, but it seems to be the results of an active cell-mediated process. We advocate use of the terms 'calcific tendinopathy' and 'insertional calcific tendinopathy' to differentiate conditions that seem to be caused by two different pathogenetic mechanisms. Better understanding of the pathogenesis is essential for development of effective treatment modalities and for the improvement of clinical outcomes.

## Competing interests

The authors declare that they have no competing interests.

## Authors' contributions

FO and NM conceived the study. FO and AGV undertook the literature search. AGV wrote the first draft. All the authors revised the manuscript, and approved the final draft.

## Pre-publication history

The pre-publication history for this paper can be accessed here:

http://www.biomedcentral.com/1741-7015/10/95/prepub
